# Idiopathic Intracranial Hypertension Animal Models and Venous Sinus Stenting: Status of Disease and Device-Focused Evidence

**DOI:** 10.3390/brainsci15101064

**Published:** 2025-09-29

**Authors:** Julien Ognard, Gerard El Hajj, Sevda Alipour Khabir, Esref A. Bayraktar, Sherief Ghozy, Ramanathan Kadirvel, David F. Kallmes, Waleed Brinjikji

**Affiliations:** 1 Department of Radiology, Mayo Clinic, First St. SW, Rochester, MN 55905, USA; elhajj.gerard@mayo.edu (G.E.H.); alipourkhabir.sevda@mayo.edu (S.A.K.); bayraktar.esref@mayo.edu (E.A.B.); ghozy.sherief@mayo.edu (S.G.); kallmes.david@mayo.edu (D.F.K.); brinjikji.waleed@mayo.edu (W.B.); 2 Univ Brest, LATIM, INSERM UMR1101, CHU Brest, 29200 Brest, France; 3 Department of Neurologic Surgery, Mayo Clinic, Rochester, MN 55905, USA; kadir@mayo.edu

**Keywords:** idiopathic intracranial hypertension, venous sinus stenting, animal models, preclinical research, translational framework

## Abstract

Background/Objectives: Idiopathic intracranial hypertension (IIH) often features dural venous sinus stenosis; venous sinus stenting (VSS) improves venous outflow and intracranial pressure, but most stents are off-label, and few are engineered for intracranial venous anatomy. The aim was to synthesize animal models relevant to IIH/VSS, catalogue stents used clinically for VSS and summarize corresponding animal data, appraise current preclinical VSS research, and propose a pragmatic preclinical evaluation framework. Methods: We performed a targeted search (PubMed, Web of Science, Scopus; through to May 2025), dual-screened the records in Nested Knowledge, and extracted the model/device characteristics and outcomes as per the predefined criteria. Results: We identified 65 clinical VSS studies; most were retrospective and used off-label carotid/peripheral/biliary stents (Precise, Zilver, and Wallstent were the most frequent). Recent dedicated systems (River, BosStent) have limited animal evidence; VIVA has GLP porcine venous peripheral data demonstrating its patency, structural integrity, and benign healing outcomes. Rodent models reproduce obesity/androgen drivers with modest, sustained ICP elevation; large animal models show the technical feasibility of in sinus implantation, but no chronic focal venous stenosis model fully mirrors the IIH condition. Conclusions: Despite broad clinical uptake, the translational underpinnings of VSS in IIH remain incomplete: most devices lack intracranial venous-specific preclinical validation, and there is no existing animal model that recapitulates both IIH biology and focal sinus stenosis.

## 1. Introduction

Idiopathic intracranial hypertension (IIH) is a syndrome involving elevated intracranial pressure (ICP), without a clearly identifiable cause. It most commonly affects young obese women and is characterized by headaches, papilledema with a risk of vision loss, pulsatile tinnitus, and often involves dural venous sinus stenosis [1]. While the exact etiology remains unclear, recent advances have begun to redefine IIH as a probable metabolic disease [2]. The transverse venous sinus stenosis observed in IIH may either be a contributing cause or a secondary effect of raised ICP. Regardless, venous outflow impairment appears central to IIH pathophysiology: elevated venous sinus pressure can reduce cerebral spinal fluid (CSF) reabsorption, exacerbating intracranial hypertension.

Venous sinus stenting (VSS) has emerged as a minimally invasive treatment for medically refractory IIH with sinus stenosis. By placing an intraluminal stent across the stenosed transverse sinus, VSS aims to restore venous drainage and reduce intracranial venous pressure. Clinically, a reduction in ICP and an improvement in the symptoms, such as headache relief and the resolution of papilledema, has been described in appropriately selected IIH patients [3]. Over the past two decades, numerous case series and cohort studies have documented the safety and efficacy of VSS in IIH, with high technical success and 6-month primary patency rates of up to 90% [4]. One contemporary concern remains the restenosis rate, which has been described as being between 15 and 20% [5]. However, the stents used in VSS are originally designed for other vascular or even non-vascular beds. A wide variety of stent devices that were originally developed for carotid, coronary, or peripheral applications, have been adapted for use in cerebral veins. These devices vary in terms of material, design, radial force, and flexibility, all of which could influence their performance in the unique environment of the dural venous sinus. In the last few years, a few stents specifically engineered for the venous sinus environment have been developed, although they remain investigational and unapproved [6]. The absence of models for venous sinus stenting significantly hinders the preclinical development of these devices and raises important translational questions in the absence of strong evidence [7]. Beyond the off-label use reported in clinical trials, how can we determine the suitability of existing stents for the dural sinus environment, and how can new venous stent technologies be evaluated prior to human use? Animal models could improve our understanding of IIH pathophysiology and serve as a testing platform for venous sinus stenting devices.

In this comprehensive review, we synthesize the current knowledge on animal models relevant to IIH and venous sinus stenting. Specifically, we aim to: First, identify and describe the animal models that replicate IIH in humans, including, if any, models evaluating VSS strategies. Second, we aim to catalog all venous stents that have been used clinically in IIH patients for transverse sinus stenting and, for each, review any published animal studies evaluating those devices, even if conducted in contexts other than VSS, such as in carotid, peripheral, or biliary stenting, to glean relevant performance metrics, such as patency, thrombogenicity, and restenosis that may translate to dural sinus applications. Third, we aim to analyze the state-of-the-art preclinical VSS research, highlighting the strengths and limitations of existing models, and examining their ability to address the needs of both VSS device testing and IIH pathophysiological research. Fourth, we propose a framework for future preclinical evaluations of VSS, grounded on scientific rationale and practical considerations.

## 2. Materials and Methods

No ethical approval was required, as the current study involved analyzing and synthesizing previously published data and information from the secondary use of deidentified electronic databases (Code of Federal Regulations, 45 CFR 46.102).

A structured and targeted literature search was conducted using Nested Knowledge^®^ (NK) AutoLit^®^ (St Paul, MN, USA), following key steps from systematic review methodology, while focusing the scope on the objectives of this work. The process combined three complementary elements.

First, we identified stent platforms used clinically for dural venous sinus stenting by reviewing human VSS studies and clinical trial registries.

Second, we performed targeted preclinical searches using these device names (including synonyms and product families), combined with animal/preclinical terms.

Third, we searched for preclinical models of elevated ICP or IIH, including those incorporating venous stenting, and reviewed the relevant paradigms.

The searches covered English-language literature, published through to May 2025, in PubMed/MEDLINE, Web of Science, and Scopus. Additional sources included hand-searching reference lists and consultation with field experts. Non-English articles were excluded.

Study screening and selection were managed within NK AutoLit^®^. Two authors (JO, GEH) independently screened the titles and abstracts, with a full-text assessment of potentially eligible articles against predefined inclusion and exclusion criteria (JO, SAK). Discrepancies were resolved by consensus. For the clinical stent identification step, we included clinical studies (excluding case reports and review articles) reporting the use of a stent in the treatment of intracranial venous sinus stenosis. For the targeted preclinical search, we included in vivo animal studies (any species) evaluating the intravascular stents used, or potentially usable, according to the previous step, for dural venous sinus stenting, which reported outcomes, such as regarding patency, neointimal formation, or thrombosis. For the animal model search, we included in vivo studies modelling elevated ICP or IIH-related states (obesity, venous hypertension, CSF dynamics alterations) with outcome measures relevant to IIH (ICP monitoring, papilledema). The data from the clinical stent literature and regulatory sources were cross-referenced with the preclinical evidence to align the findings on device use, model fidelity, and the translational relevance.

A predefined data extraction form was employed. Any discrepancies or conflicts were resolved through discussion and consensus. From each study, key data were extracted, including animal species/strain, experimental manipulations, duration of the model, and reported effects on ICP, CSF production or absorption, venous sinus anatomy, and neurological signs. For device evaluations, we noted the stent brand/model, stent material and design, animal model, and any quantitative outcomes.

We tabulated the identified animal models of IIH and of VSS and compared them qualitatively. In analyzing the “state of the field,” we emphasized whether the current models truly recapitulate the human disease’s mechanistic contributors and whether they have been used to test interventions. For stent devices, we synthesized the findings across the animal studies to derive general insights into their performance in different conditions, and the implications for intracranial venous use.

## 3. Results

### 3.1. Animal Models: IIH, Elevated ICP, Venous Stenting

Developing an animal model of IIH requires reproducing the chronically elevated ICP and key clinical features, such as papilledema and headache surrogates, without a mass lesion. Recent efforts have yielded promising models by targeting the known risk factors of IIH: obesity and female hormonal milieu [8]. Below, we describe these models, including their creation, the extent to which they mimic human IIH, and their limitations.

#### 3.1.1. Diet-Induced Obesity Rodent Models

Given the strong association between obesity and IIH, researchers have attempted to induce intracranial hypertension in rodents via a high-fat diet and weight gain. Notably, Wardman et al. developed a rat model by feeding female Wistar rats a hypercaloric high-fat diet for 21 weeks [9]. These obese rats exhibited a 65% elevation in ICP compared to lean controls, a significant and sustained increase. For context, if the baseline ICP in lean rats is approximately 5 mmHg, a 65% rise would correspond to ~8–9 mmHg, approaching the upper limit of normal for rodents. This elevation was accompanied by a 50% increase in CSF outflow resistance, suggesting that obesity may impair CSF drainage, potentially due to elevated venous sinus pressure or lymphatic dysfunction. Importantly, the obese rats did not show an increased CSF production rate or major changes to choroid plexus gene expression in that study. This mirrors some IIH patients in whom the primary issue appears to be reduced CSF absorption rather than overproduction [10].

An earlier study by Uldall et al. similarly demonstrated that obese Zucker rats (a genetic obesity model) have chronically higher ICP than lean littermates [11]. Over 4 weeks of continuous monitoring, obese Zucker rats had significantly elevated ICP on nearly all of the days (on average about a 1.5–2 mmHg higher ICP than lean rats). They also displayed upregulation of choroid plexus aquaporin-1 (AQP1) at both mRNA and protein levels. AQP1 is involved in CSF secretion; increased AQP1 could mechanistically contribute to increased ICP by enhancing CSF production. These findings position obese rodent models as potential analogues of IIH, as they replicate the association between obesity and elevated ICP and point toward a possible choroid plexus interaction like that proposed in human IIH [12,13].

Despite successes, pure obesity models have limitations. In both the high-fat-diet rats and Zucker rats, the magnitude of ICP elevation is modest, with no reports of overt papillary edema or neurological impairment. Additionally, key features of IIH, such as sex bias and venous sinus stenosis, are not replicated in these models. Recently, Jensen et al. reported that Zucker obese rats, despite marked obesity, maintained normal ICP unless exogenous testosterone was administered [14]. In their study, obese rats demonstrated undisturbed CSF testosterone levels and no changes in ICP or CSF dynamics.

#### 3.1.2. Androgen Excess Models

Motivated by the observation that IIH patients often exhibit a polycystic ovary syndrome-like androgen profile, including elevated testosterone in women [15], researchers have tested whether androgen excess can raise ICP (by administering exogenous testosterone to lean female rats for 28 days) [9]. This intervention caused a 55% increase in ICP, a rise comparable in magnitude to that observed with obesity. Notably, unlike diet-induced obesity alone, testosterone administration also caused an ~85% increase in the CSF secretion rate. The testosterone-treated rats showed evidence of upregulated choroid plexus transport, with increased activity of the Na^+^-K^+^-2Cl^−^ cotransporter (NKCC1), a key driver of CSF secretion [16]. NKCC1 overactivity logically leads to elevated CSF production, contributing directly to increased ICP. These results strongly suggest that androgen excess can independently elevate ICP via stimulating CSF production. In any case, the combination of diet-induced obesity and androgen excess seems to recapitulate both elements of IIH: increased resistance to CSF outflow (from obesity) and increased CSF production (from androgens).

#### 3.1.3. CSF Hypersecretion and Absorption Models

Aside from the use of models, some older attempts tried to pharmacologically induce intracranial hypertension. One hypothesis was that vitamin A excess, known to cause IIH in humans [17], might create an IIH-like state in animals. However, experiments in rats yielded paradoxical results: high-dose vitamin A actually decreased CSF pressure in one study, likely by increasing CSF absorption [18], possibly due to the upregulation of the arachnoid granulation function.

On the other hand, direct CSF dynamics manipulation models have been used to study raised ICP in a controlled manner [19]. For example, implanting intraventricular infusion pumps or induced CSF outflow obstruction have been used to acutely elevate ICP in rodents [20]. Although these models do not replicate idiopathic disease, they provide a platform for testing the physiological consequences of elevated ICP. In a pig model, investigators inserted an inflatable balloon into the subarachnoid space to partially occlude CSF outflow, successfully creating a sustained ICP elevation of approximately 30 mmHg for several hours [21,22]. Such approaches demonstrate feasibility, but are acute and do not reproduce the chronic remodeling seen in IIH.

#### 3.1.4. Venous Outflow Impairment Models

Since venous sinus stenosis is a hallmark of IIH, an ideal animal model would reproduce a similar venous condition. Achieving this chronically remains challenging. Acute intracranial venous hypertension can be modeled by occluding venous sinuses, such as the transverse sinus or jugular veins, endovascularly. For example, cerebral venous sinus thrombosis (CVST) models in rats involve injecting thrombogenic materials, such as kaolin or using photochemical agents, in regard to the superior sagittal sinus to induce thrombosis [23,24]. However, this approach results in a marked rise in ICP, along with venous infarction and neurological injury. In one such model, the ICP rose by >200% within hours, accompanied by brain edema [25]. While useful for studying venous stroke, CVST models are too severe and acute to reflect the chronic, idiopathic nature of IIH.

A more subtle and controlled venous narrowing is harder to achieve, but has been attempted. Lavoie et al. [22] developed an intracranial venous hypertension model in large animals (three swine and two baboons) by mapping the cerebral venous anatomy and inducing the stepwise partial occlusion of dural venous outflow, using either bareplatinum coils or autologous blood clots. Angiography and venography revealed that baboons primarily drain through the internal jugular veins, while pigs rely mainly on a paraspinal venous plexus. Notably, both species possess a petrosquamous sinus that serves as a collateral drainage pathway. In baboons, the direct continuity between the dural sinuses and the jugular vein allowed for a percutaneous retrograde catheterization; however, they noted the absence of this connection in pigs, which necessitated a transcranial approach in order to reach the target sinus. The stepwise partial occlusion of the sinus successfully elevated the intracranial pressure in both models without compromising survival, and all the animals remained neurologically intact six months post-procedure. These findings demonstrate that reproducible venous outflow blockades can be achieved in both species.

#### 3.1.5. Models of Dural Sinus Venous Stent Implantation Without Venous Outflow Impairment

Several large animal studies have shown that full-scale human venous stents (or stent-derived devices) can be deployed safely in non-stenotic dural sinuses, providing a clean platform to study device biomechanics, endothelialization, and long-term patency, without the confounder of flow-limiting disease.

Among large animals, the ovine model has become a foundational platform for preclinical research on endovascular brain–computer interfaces (BCIs), due to its anatomical and physiological resemblance to the human cerebral venous system [26]. Oxley et al. first detailed a method for cerebral venography in sheep, demonstrating the model’s suitability for catheter-based neurovascular interventions [27]. As summarized before, the venous anatomy of sheep aligns with that of humans and supports the evaluation of implantable neural devices or venous stents [28]. The description of this model and its previous application to assess the histological integration of BCIs remain relevant for our purposes, even if deployed in a healthy environment, involving 185 sheep, as reported in the literature [26]. However, several reports also highlight important technical limitations, including substantial inter-individual variability and some anterior sinus segments that can be of very small caliber, which makes selective catheterization technically demanding [27].

Swine models are technically more challenging, because, as stated, porcine dural sinuses primarily drain into a paraspinal plexus, limiting percutaneous access [29,30]; nonetheless, some researchers have attempted, with success, percutaneous catheterization of the superior sagittal sinus (SSS) in Yorkshire pigs. Pasarikovski et al. navigated a 2.7 F OCT catheter into the sinus (average luminal diameter = 3.14 mm, which is at least twice as small as the human diameter) and recorded high-resolution intraluminal images, without complications, indicating that 3–4 mm self-expanding stents could be tested in vivo, albeit with careful sizing to avoid the need for oversizing-related remodeling [31].

Taken together, current evidence supports the ovine SSS as the most practical and anatomically relevant venue for first-line preclinical testing of dural sinus stents: Even if the diameter of adult human venous vessels is approximately three to five times that of sheep vessels at comparable positions (transverse sinus 5–7 mm vs. 2–3 mm, human vs. sheep [26,28,32]), chronic implantation for ≥6 months is feasible with reliable patency, and the tissue responses are quantifiable. To mitigate ovine model constraints, investigators commonly perform pre-procedural venographic imaging to select animals with a favorable anatomy, consider an open jugular cut-down method to facilitate sheath placement, use lower profile delivery systems adapted to ovine dimensions, or choose porcine models when testing stents intended for human calibers.

To date, there is no well-established chronic dural venous stenosis animal model that mirrors pure venous stenosis without thrombosis and that has been used for testing stents. This remains a key gap in the research. Developing such a model, potentially through the use of an adjustable external stent or gradual occlusive device placed on the sinus of a large animal, would be of interest for directly testing venous sinus intervention devices.

#### 3.1.6. Models of Healthy Peripheral Veins (Without Being Intracranial nor Having Venous Outflow Impairment)

Many peripheral vein models can be applied across species for various research purposes [33]. Here, we summarize the platforms that may offer advantages for dural sinus research: they provide vessel diameters (8–14 mm) that closely match the human transverse sinus, exhibit venous flow profiles and wall composition that may be assumed to approach intracranial sinuses, and allow for long-term survival studies that can be used to assess endothelialization and neointimal thickness, without confounding disease influence.

In sheep, two complementary paradigms dominate. One comprises a deployment in the common iliac vein. A report using this mode to study a nitinol stent demonstrated 100% implant success, the absence of migration, and complete endothelial coverage by 90 days. Histological analysis confirmed that the early inflammatory infiltrate subsided by six months, while the luminal loss remained <10% at the one-year follow-up [34,35]. The second ovine model involved placement in the inferior vena cava (IVC). For example, a study using this model demonstrated that the sheep IVC tolerates large nitinol frames and allows for the systematic manipulation of certain design variables, such as the ring spacing and overall length [36]. Design–response studies have exploited this advantage. Adjusting the axial gap between stent rings in the sheep IVC revealed that wider spacing accelerates endothelial coverage and reduces the intimal burden, implying that the metal-to-vein ratio is a modifiable determinant of venous healing [37].

In parallel, porcine studies have interrogated the effects of stent oversizing: Jugular and iliac veins fitted with self-expanding nitinol stents that were 120–216% of the native diameter remained patent; however, the neointimal area increased linearly with the degree of oversizing (r ≈ 0.8), and sporadic migration occurred when the device–wall apposition was insufficient [38]. The internal mammary vein porcine model was recently chosen for use in a study, due to its relative accessibility, consistent diameter, and size similarity to the human transverse sinus [39].

In “small animal” studies, the New Zealand white rabbit offers a convenient paired control framework, as each animal’s right and left internal/external iliac veins (3–4 mm) can receive different test devices, allowing for a within-subject comparison of the neointimal response and patency [40]. This model is widely used in device development [41].

A key limitation of peripheral vein models (and of implanting venous sinus stents into the cerebral sinuses of otherwise healthy animals) is that they do not reproduce the focal extrinsic compression and the rigid dural enclosure characteristic of idiopathic intracranial hypertension. Consequently, stent behavior observed in peripheral veins or in the cerebral sinuses of healthy animals may not predict device performance when a sinus is subject to elevated intracranial pressure, local dural collapse, or bone–sinus interactions, as seen in IIH [42]. The wall structure of peripheral veins also differs fundamentally from that of dural venous sinuses. Peripheral veins possess a conventional three-layered wall and often contain venous valves. By contrast, dural venous sinuses are endothelium-lined channels, formed between layers of dura mater; they lack regular valves and do not have typical muscular tunica media [42]. Also, smaller animal models have a small vessel size, which necessitates the design of custom-made stents that may not adequately reflect the geometry, radial force, or material properties of clinical venous stents. They have also been shown to exhibit exaggerated endothelial proliferation and a neointimal response due to both size-related flow differences and higher metabolic rates, which bears little resemblance to the complex fibrocellular and thrombotic responses observed in humans [43]. In cases involving the use of elastic arteries, such as the aorta, as surrogate implantation sites, important biases are introduced, since these vessels are less prone to injury than muscular veins [44]. Nevertheless, these models serve as valuable first-stage testing sites, enabling the early evaluation of safety, patency, and endothelialization profiles, before advancing to the more demanding cranial venous sinus or disease-specific models.

### 3.2. Venous Sinus Stents Used in IIH Patients and Corresponding Animal Data

Across our review, 65 studies included patients undergoing venous sinus stenting, of which most of the reports were retrospective (51/65, 78.5%) and were conducted primarily in the USA (38/65) and China (10/65). Among single-device case series, off-label stents were mostly used. These include a diverse array of devices originally developed for other vascular territories, such as carotid artery stents, peripheral vascular stents, and biliary stents, each with distinct design characteristics. Precise was the most common stent used (16 studies, 765 patients), followed by Zilver (11 studies, 249 patients), and Wallstent (7 studies, 158 patients). Dedicated intracranial venous systems were used more recently (2024–2025): River (1 study, 39 patients) and BosStent (2 studies, 39 patients) (Table 1).

In the early 2000s, when Higgins et al. first pioneered VSS for IIH, self-expanding stainless-steel Wallstents were used [98]. Over time, nitinol (nickel–titanium alloy) stents largely replaced other alloys due to their better flexibility and biocompatibility. The Precise stent emerged as a popular choice and is still widely used. The diversity of the stents on the market reflects both their availability and operator preference, as well as the evolution of stent technology. This section provides a comprehensive catalog of the stents available and summarizes the available preclinical data gained from animal studies, where applicable, even when those studies were conducted for the device’s original (non-neurovascular) indication (Table 2).

#### 3.2.1. Precise^®^ Pro RX Carotid/Peripheral Stent System (Cordis, Santa Clara, CA, USA)

The Precise stent is a nitinol self-expanding carotid stent, with an open-cell design. The FDA premarketing testing involved both acute and chronic studies. Chronic studies were conducted on canine models, using a similarly designed stent (S.M.A.R.T stents), with successful deployment, complete endothelialization, and patency, without inflammation, for up to 12 months. Acute studies were performed on porcine models and focused on evaluating stent performance and mechanical characteristics, ease and precision of placement, and migration rates. The outcomes across all of the parameters were rated as excellent, good, or acceptable [104].

The Precise stent has been evaluated using porcine models as a “reference” stent [39,103]. In a study, 14 Precise stents were implanted in minipig common carotids for 4 weeks and yielded no stent thrombosis. Quantitatively, the mean in stent stenosis by area was ~34% at 28 days. Histology showed the stents were well endothelialized, with minimal inflammation. The maximal neointimal thickness was about 0.5 mm [103]. In a study involving domestic pigs using the internal mammary vein, Precise stents served as a control and led to a significant increase in the venous diameter post-deployment [39]. Patency in these animal implants was 100% at all of the time points. Precise stents achieved favorable endothelialization in high- and low-flow settings [139]. Clinically, it was the predominant stent (80%) used in a Chinese IIH case series, chosen for its trackability [80].

#### 3.2.2. Protégé^®^ RX Carotid/EverFlex™ Peripheral Stent System (Medtronic Vascular Inc., Plymouth, MN, USA)

Protégé is a nitinol self-expanding carotid stent, with a closed-cell or hybrid design. While specific publications are sparse, the Protégé stent (also known as EverFlex in regard to peripheral use) has undergone standard preclinical testing. According to the FDA summary, the Protégé stent was evaluated as a peripheral device in pig iliac, carotid, and subclavian arteries, as well as in carotid and iliac arteries in canine models. The studies reported no acute thrombosis and an acceptable intimal response [106]. Bench studies indicate that the Protégé stent has slightly higher radial stiffness, but lower flexibility than the Precise stent [105].

#### 3.2.3. Carotid/Peripheral WALLSTENT^®^ Endoprosthesis (Boston Scientific Corp., Marlborough, MA, USA)

The Wallstent is a self-expanding braided stent, made of a cobalt–chromium + iron–nickel–molybdenum (originally stainless steel) alloy, containing an enhanced radiopaque tantalum core, with a closed-cell design. It was one of the first stents used for venous sinus stenosis [98]. Wallstent has been approved by the FDA for treating central venous and iliofemoral venous outflow obstructions. However, no animal testing was reported in the FDA’s “Summary of Safety and Effectiveness” [140]. Across iliac, carotid, and jugular models in pigs, dogs, sheep, and rabbits, the Wallstent consistently showed easy deployment, long-term patency, low thrombogenicity, modest neointimal hyperplasia, high endothelialization, and low inflammation scores [114,116,117,119,121,125]. In a sheep iliac vein model, the Wallstent achieved complete endothelialization, with no thrombosis, and a stenosis rate of approximately 22.5% at 30 days and 32% at 90 days. In swine carotid studies, a prototype nitinol braided stent demonstrated comparable outcomes to laser-cut stents [103]. However, the stainless steel Wallstent may induce greater inflammation, as stainless steel contains more nickel than nitinol [141]. Mechanical limitations have also been reported in some models [142]. In rabbit and swine studies, Wallstent implantation significantly reduced local vessel compliance and increased stiffness and impedance, which may predispose the vessel to restenosis [123,130]. In a retrospective study of IIH patients, 49 individuals received Wallstents in their dural sinuses, with outcomes comparable to those reported in other case series [68]. In this study, the authors reported that while the stents offer some flexibility, they can be more challenging to position with precision and their delivery to the site of venous stenosis is more difficult, especially in cases with severe stenosis or high-riding jugular bulbs.

#### 3.2.4. Zilver^®^ 518/Flex Vascular/Biliary Stent (Cook Medical LLC, Bloomington, IN, USA)

Zilver is a nitinol self-expanding stent, originally designed for use in biliary and peripheral arteries. The Zilver 518 and Zilver Flex stents have been frequently used in IIH, often described as “biliary stents” in reports, due to the use of the biliary self-expanding stent. Animal data on the use of this stent is more extensive, mostly due to the development of the Zilver paclitaxel (PTX) drug-eluting peripheral stent. In pigs, over 400 bare Zilver stents were tested in iliac and femoral arteries, as part of the FDA approval process [115]. The results showed no safety issues and “complete vessel healing without negative sequelae” by 1–2 months. Specifically, there were zero stent thromboses in 180 pigs. Histology confirmed full endothelial coverage and only a mild inflammatory reaction to nitinol. Neointimal hyperplasia in the pig’s superficial femoral artery was moderate. The FDA summary noted that the biological response was comparable to that observed with other bare nitinol stents, and the addition of paclitaxel did not appreciably alter the vascular response. Clinically, Zilver stents have one of the longest track records in venous sinus applications. Many centers favor the Zilver stent that is 8 mm in diameter, due to its ready availability and high flexibility. Reported restenosis rates for the Zilver stent in IIH are approximately 10–15%, which falls within the range described in the literature. Notably, the Zilver stent employs an open-cell design (although with smaller cells than the Precise stent), which may allow tissue to prolapse in very soft venous walls. Although no direct animal venous data exist, a swine TIPS model (portal–caval shunt) showed poor patency with the use of bare Zilver stents, attributed to aggressive pseudointimal formation in the low-flow hepatic environment [111]. This limitation was solved clinically by using covered stents for the TIPS procedure. By analogy, flow in dural sinuses is higher than in the TIPS procedure [143], which may also have contributed to the generally good patency of stents like the Zilver stent in this setting.

#### 3.2.5. Acculink^®^ Carotid/Xact^®^ Carotid/Xpert^®^ Self-Expanding Stent System (Abbott, IL, USA)

Acculink and Xact are nitinol, self-expanding carotid stents, with Acculink featuring an open-cell design and Xact a closed-cell design. Both are popular carotid stents and have also been used off-label in the treatment of IIH [64]. FDA approval studies of the Acculink carotid stents were performed using a non-atherosclerotic swine model. Implanted stents did not elicit acute or chronic thrombosis, adverse inflammatory reactions, or excessive neointimal proliferation. Patent lumen was maintained immediately after deployment and at all re-look time points [132]. In fact, one bench study found Acculink had the largest cell area of carotid stents (11.5 mm^2^) [144], implying lower coverage. This may translate to a reduced intimal response, but also to less effective scaffolding of the vessel wall. No published animal data is available; testing for the Xact stent was conducted as part of the FDA’s approval studies, demonstrating good outcomes in porcine carotid and iliac arteries in terms of safety and performance [133]. The Xpert stent is a thin-strut, nitinol stent, used primarily for peripheral stenting [145]. An ex vivo study on explanted porcine arteries provides a computational hemodynamic analysis [146].

#### 3.2.6. LifeStent/LifeStar^®^ Vascular Stent System (Bard Peripheral Vascular Inc., Becton, Dickinson and Company, Tempe, AZ, USA)

LifeStent is a nitinol self-expanding stent, originally designed for use in lower extremity peripheral artery disease [147]. High-radial-force stents like LifeStent were tested in pig iliofemoral arteries. A notable finding is that the chronic outward force (COF) may influence intimal hyperplasia. A comparative study is currently underway to evaluate whether high-COF stents like LifeStent are associated with greater restenosis compared to lower-COF designs (such as BioMimics 3D) [148,149]. As part of the FDA approval process, LifeStents were implanted in various peripheral vascular beds, including the femoral and iliac arteries, demonstrating high patency rates and minimal vessel wall response to the stent [134]. Previous animal studies have suggested that regions of low wall shear stress, which are often associated with stiffer stents, are correlated with increased neointimal formation [150]. The LifeStent otherwise had excellent patency in SFA clinical trials (RESILIENT trial: ~80% at 3 years) [151]. The LifeStent is the 5F low-profile design based on the LifeStar stent.

#### 3.2.7. Casper™/Roadsaver^®^ Carotid Artery Stent System (Terumo MicroVention, Tustin, CA, USA)

The Casper/Roadsaver stent, is a dual-layer, braided-wire carotid stent, combining an outer ∼180 μm wire frame with an inner ∼42 μm mesh (pore size 0.15–0.20 mm^2^), linked by ∼45 μm connectors. The four-wire-layer structure yields a mean thickness of ∼1.7 mm [152]. This second-generation carotid stent is designed to prevent plaque prolapse through the use of an inner woven mesh. As part of FDA approval, a chronic porcine study implanted Casper stents in carotid and subclavian arteries of 12 pigs, with sacrifices at 1, 3, 6, and 12 months, with widely patent lumens and complete neointimal incorporation in most stents [135]. No other animal studies have been published in regard to this stent. Clinically, the Casper stent has been used in IIH, including in a published case series by Belachew et al., who compared 10 patients treated with Casper to 5 treated with the Precise stent [56]. Both groups had 100% technical success and no periprocedural thromboses; at a mean duration of 16 months, all of the stents remained patent. The nitinol material, the braided structure, and the low profile offers high flexibility in order to navigate the tortuous transverse–sigmoid junction [56].

#### 3.2.8. Other Stents

Intracranial artery stents, such as Enterprise (Enterprise^®^ Vascular Reconstruction Device (VRD) (Codman Neuro, part of Integra LifeSciences, Raynham, MA, USA)) and Solitaire AB (Solitaire™ AB Revascularization Device (Medtronic Neurovascular, Irvine, CA, USA)) have been used in a small number of IIH cases as venous sinus stents. These are neurovascular devices with smaller diameters (4.5–6 mm) and were likely selected for use in cases involving very narrow or distal transverse sinus anatomy. Animal data on the use of Solitaire AB (closed-cell design) in swine subclavian and carotid arteries demonstrated excellent device performance, with no device-related acute thrombosis [137]. Enterprise (closed-cell intracranial stent) was tested in swine carotid arteries and was found to have high flexibility and a low radial force [136]. There are no specific reports on Solitaire used chronically as a stent in animals, since it became a retriever.

#### 3.2.9. Dedicated Stents

The River stent (River™ Venous Stent System (Serenity Medical Inc., Redwood City, CA, USA)) is the first stent tailored for cerebral venous sinus stenosis. It employs a tapered nitinol stent (larger diameter at proximal end), with high flexibility and a lower metal surface area to minimize jailing of cortical veins [153]. Patsalides et al. reported that in the first human case series involving 39 IIH patients, the River stent achieved 100% technical success, no intra-op thrombosis, and a significant sinus pressure gradient reduction, with >90% of patients experiencing improvements at 6 months [75].

Another dedicated device is the BosStent (BosStent^®^ Venous Stent System (Sonorous NV, Niel, Belgium)). No FDA data are available on this stent. While no animal data have been published on the use of this stent, an early clinical case series has been reported by Mendes-Pereira et al. [76] and Consoli et al. [154]. In this case series, they reported that its braided structure facilitates cell navigation and provides a stable base for jailing, while the dedicated 5 Fr resheathable delivery system enabled smooth deployment in tortuous anatomies. VasoCT confirmed optimal apposition, without the need for post-deployment angioplasty in most cases.

The Viva (VIVA™ Venous Sinus Stent System (V-Flow 21 Ltd., Or Akiva, Israel)) [39] is the only venous sinus stent that has published preclinical data on its use, derived from a Good Laboratory Practice (GLP)-compliant porcine venous implantation model. No thrombus formation was observed. CT venography confirmed vessel patency, the absence of stent migration, and complete structural integrity. Histopathology revealed a mild, expected foreign body reaction at 30 days that had been resolved by 180 days, consistent with normal healing, along with an increased luminal diameter and a reduced wall thickness at 180 days.

The overall takeaway is that most stents used in IIH have performed well in the respective animal tests involving other vascular beds. Differences arise in the degree of neointimal hyperplasia: Designs with a higher radial force (LifeStent, possibly Wallstent) tend to provoke a bit more of a tissue response, whereas newer carotid stents (Precise, Casper) strike more of a balance between support and minimal vessel injury. None of the stents have shown acute thrombogenicity when used in animal models. This aligns with clinical experience that in stent thrombosis in VSS is quite rare (especially with dual antiplatelet therapy peri-procedurally).

One must consider that animal studies usually involve healthy vessels; IIH venous sinuses may have a diseased endothelium or external compression, contributing to stenosis. Still, animal data gives us confidence that these stents are not likely to fail due to intrinsic design flaws. The main long-term issue to watch is restenosis as a result of intimal hyperplasia. Animal models suggest a plateau in neointimal growth by 3 months for nitinol stents [103]. If a stent remains widely patent at that point (with ~20–30% narrowing), it will likely remain stable in the long term. Most clinical restenoses after venous stenting present within the first year and are frequently located at stent margins [155]. Animal studies mirror this pattern, with the greatest neointimal growth at the ends of stents (likely due to flow perturbation). Contributing mechanisms may include raised ICP or trans-sinus pressure gradients, ongoing extrinsic compression, lax sinus walls, or a delayed/unregulated endothelial healing response [43,156,157].

## 4. Discussion

We report that most stents currently used clinically for VSS in IIH are off-label adaptations of devices designed for other vascular territories, with only two purpose-built intracranial venous stents having been developed in recent years; both of which remain investigational [75,76]. Although significant human clinical experience exists now, with more than two thousand patients treated (using more than 15 devices), these stents have been evaluated primarily under high-flow conditions, with fewer studies in low-flow settings and none involving true intracranial venous anatomy within animal models. Surprisingly, no animal testing was found for the recently developed and available dedicated intracranial venous stent. Only one VSS-specific device, still in development, has undergone preclinical testing in an animal venous model (internal mammary vein) [39]. And yet, as demonstrated, although a comprehensive animal framework encompassing both IIH and venous sinus stenosis has not been fully established, suitable platforms are available to evaluate device behavior and integration. Notably, BCI stents, such as the Stentrode^®^ Endovascular Neural Interface (Synchron Inc., Brooklyn, NY, USA), designed for implantation in the superior sagittal sinus of ALS patients, have been extensively evaluated in ovine SSS models [26].

Historically, progress in the understanding of venous sinus stenting has been hindered by a lack of animal models that accurately mirror the key features of idiopathic intracranial hypertension. Traditional models of intracranial hypertension, such as acute CSF infusion, traumatic brain injury, or hydrocephalus, do not replicate the specific venous sinus narrowing characteristic of IIH. Moreover, the dural venous sinuses of small animals are too small to accommodate stenting, while larger animals rarely develop IIH-like conditions naturally. Bringing together the findings from Section 3.1 and Section 3.2, we can evaluate how well the current animal models serve the needs of VSS research. There are two intertwined aspects: modeling the disease (IIH) itself and modeling the intervention (venous stenting).

Large animal studies, predominantly in ovine and swine, have demonstrated that venous sinus catheterization and stenting is technically feasible in a biologically relevant setting. Such studies help build operator experience and identify any acute anatomical challenges. Importantly, stent healing observed in pig vasculature has provided confidence that modern self-expanding stents are unlikely to thrombose or cause a fulminant reaction when deployed in the venous sinus. Another strength is that large animals can accommodate device iterations; manufacturers can test different stent lengths, diameters, or flexibility profiles in a pig model to evaluate its conformability to the sinus and identify risks, such as kinking or migration, before finalizing the design.

Nonetheless, no animal model to date spontaneously develops focal dural venous sinus stenosis, resembling that seen in IIH. This is of importance because the biomechanical effect of a stenosis (venous pressure gradient) and the remodeling of the sinus wall are not replicated. Consequently, the current models cannot assess how intracranial pressure responds to stent placement in the context of true idiopathic venous narrowing, as opposed to deployment in a normal, non-stenotic vessel. A potential promising method is endovascular radiofrequency (RF) ablation, which has emerged as a reliable, controllable, and minimally invasive technique for inducing venous stenosis and subsequent thrombosis in large animals. RF ablation creates venous wall and luminal changes that more closely resemble human pathology, including intimal thickening and smooth muscle proliferation [44,158]. Various models of cerebral venous sinus thrombosis (CVST), including photo-thrombosis, electrocoagulation, the injection of thrombogenic substances, or direct clot injection, have also been developed. However, these models tend to be too acute, often result in parenchymal injury, and do not replicate the chronic, compensated pathophysiology characteristic of IIH [24,159]. This research gap highlights that the causal role of venous stenosis in maintaining ICP or the full physiological response to stenting, has not been validated in animal models. Instead, we rely on human data and fluid dynamics models to assume that relieving stenosis lowers ICP [95].

Chronically elevated ICP can cause pain and neurological issues in animals, raising ethical issues, unless carefully managed. Continuous ICP monitoring in awake animals is also technically difficult. Most ICP measurements in pigs have been performed acutely under general anesthesia, which itself alters ICP and CSF dynamics: isoflurane can increase cerebral blood flow and ICP, while propofol tends to reduce them. These effects confound long-term ICP assessment in large animal models. Rodents can be fitted with telemetric ICP probes, and some laboratories have successfully implemented these devices in obese rat models, enabling the application of long-term intracranial pressure.

Animal studies of stents typically focus on vessel patency and histology, but in the context of IIH, other relevant outcomes, namely ICP reduction and papilledema resolution, may be of interest, particularly as an adapted model can also be designed.

Sheep have a relatively less active extrinsic coagulation pathway than humans, with higher platelet counts, but reduced platelet activation and biomaterial reactivity. While both sheep and pigs share broadly similar intrinsic coagulation characteristics as humans, pigs more closely resemble the human profile, except for their higher platelet counts and stronger platelet aggregation. These interspecies differences mean that histological outcomes from preclinical stent studies must be interpreted with caution [160]. They also highlight the value of using appropriate models to test different antithrombotic regimens on venous stent patency, noting that antithrombotic therapy is routinely given to patients after venous sinus stenting and, thus, comparable conditions exist between preclinical and clinical settings. To date, however, such studies have not been performed, and clinical protocols remain those extrapolated from arterial stenting experience.

To guide model selection and refinement, several key attributes are considered [161]. Predictive validity assesses the model’s ability to forecast human outcomes, while face validity focuses on observable similarities in the symptoms and disease presentations. Construct validity ensures that the underlying biological mechanisms align with those in humans. The models must also demonstrate reproducibility through the generation of consistent results, sensitivity by detecting subtle changes, and specificity by accurately simulating a particular disease without extraneous interference [162]. Ethical considerations require adherence to guidelines that minimize suffering and employ the principles of replacement, reduction, and refinement. Practicality influences the feasibility and cost effectiveness of conducting research, whereas time efficiency ensures that disease progression occurs within an acceptable timeframe. Relevance requires that the model authentically reflects the complexity of the human condition, and scalability allows for the expansion of experiments to achieve sufficient statistical power. Given the above analysis, we propose a framework for evaluating VSS devices in preclinical models (Table 3).

## 5. Conclusions

Venous sinus stenting (VSS) has emerged as a promising therapy for idiopathic intracranial hypertension (IIH), yet its translational underpinnings remain unclear. Rodent models mirror obesity/hormonal factors without focal sinus stenosis, and large animal studies enable deployment testing, but do not involve chronic intracranial hypertension. Clinically, most stents are off-label; only two venous sinus-dedicated systems are under investigation, and thousands of patients have been treated, yet preclinical testing has largely occurred in regard to high-flow beds, with only a few low-flow studies and none involving true intracranial venous anatomy. We found no animal testing for the recently developed dedicated devices, and only one VSS-specific platform (still in development) has been evaluated through the use of an animal peripheral venous model. And yet, in sinus large animal platforms exist: BCI devices show durable deployment and integration in the ovine superior sagittal sinus, demonstrating feasibility. Applying previously studied models and developing robust, ethically sound, and translationally relevant animal models is essential to advance device development and the pathophysiological understanding of VSS in IIH.

## Figures and Tables

**Table 1:** Venous sinus stents used clinically for venous sinus stenosis in idiopathic intracranial hypertension. (If a study appears more than once in the table, the specific number of stents used in each category is detailed in the Notes column.).

Author [Ref.]	Year	Design	Country	Number of Patients	Notes
**Precise (Cordis) (exclusively)**				**Total Patients: 765, Studies: 16**	
Touzé R et al. [45]	2021	Retrospective	France	16	
Schwarz et al. [46]	2021	Retrospective	USA	8	
Garner et al. [47]	2021	Retrospective	USA	81	
Shazly et al. [48]	2018	Retrospective	USA	3	Precise (n = 3) or Zilver (n = 3)
Liu et al. [49]	2017	Prospective	USA	4	Precise (n = 4), Protégé (n = 6), Wallstent (n = 2)
Satti et al. [50]	2017	Retrospective	USA	43	
Zehri A et al. [51]	2022	Retrospective	USA	10	
Martinez-Gutierrez et al. [52]	2022	Prospective	USA	53	Size used: 8 mm × 40 mm
Raynald [53]	2023	Retrospective	China	121	
Buhbut [54]	2024	Retrospective	Israel	37	Size used: 7 mm × 40 mm; 8 mm × 40 mm
Yang [55]	2022	Prospective	China	121	
Belachew [56]	2021	Retrospective	Switzerland	5	Casper (n = 10) or Precise (n = 5)
Dinkin et al. [57]	2017	Prospective	USA	13	
He [58]	2024	Retrospective	China	172	
Wang [59]	2025	Prospective	China	74	
Lee KE et al. [60]	2021	Retrospective	USA	4	Precise (n = 4), Zilver (n = 7), Protégé (n = 2), or Fielder (n = 1)
**Zilver (Cook Medical) (exclusively)**				**Total Patients: 249, Studies: 11**	
Shields et al. [61]	2019	Retrospective	USA	42	
Cappuzzo et al. [62]	2018	Retrospective	USA	18	8 mm × 60 mm
El Mekabaty et al. [63]	2017	Retrospective	USA	31	Biliary, 7–9 mm × 30–60 mm
Kumpe et al. [64]	2017	Retrospective	USA	39	
Ducruet et al. [65]	2014	Retrospective	USA	30	
Fields et al. [66]	2013	Retrospective	USA	15	518, 10 mm
Albuquerque et al. [67]	2011	Prospective	USA	18	
Bilgin [68]	2023	Retrospective	USA	28	
Hendrix [69]	2022	Retrospective	USA	18	
Shazly et al. [48]	2018	Retrospective	USA	3	Precise (n = 3) or Zilver (n = 3)
Lee KE et al. [60]	2021	Retrospective	USA	7	Precise (n = 4), Zilver (n = 7), Protégé (n = 2), or Fielder (n = 1)
**Wallstent (Boston Scientific) (exclusively)**				**Total Patients: 158, Studies: 7**	
Asif et al. [70]	2018	Retrospective	UK	41	
Matloob et al. [71]	2017	Retrospective	UK	10	
Bilgin [68]	2023	Retrospective	USA	53	Wallstent (n = 53) or Zilver (n = 28)
Handzic [72]	2025	Retrospective	Canada	15	Wallstent (Boston Scientific)
Larson [73]	2020	Retrospective	USA	16	
Lenck et al. [74]	2017	Retrospective	France	21	
Liu et al. [49]	2017	Prospective	USA	2	Precise (n = 4), Protégé (n = 6), Wallstent (n = 2)
**Protégé (Medtronic) (exclusively)**				**Total Patients: 8, Studies: 2**	
Liu et al. [49]	2017	Prospective	USA	6	Precise (n = 4), Protégé (n = 6), Wallstent (n = 2)
Lee KE et al. [60]	2021	Retrospective	USA	2	Precise (n = 4), Zilver (n = 7), Protégé (n = 2), or Fielder (n = 1)
**Casper (Terumo) (exclusively)**				**Total Patients: 10, Studies: 1**	
Belachew [56]	2021	Retrospective	Switzerland	10	
**River stent (Serenity Medical), venous specific (exclusively)**				**Total Patients: 39, Studies: 1**	
Patsalides [75]	2025	Prospective	USA	39	River stent (Serenity Medical)
**BosStent (Sonorous), venous specific (exclusively)**				**Total Patients: 39, Studies: 2**	
Mendes Pereira et al. [76]	2025	Retrospective	Canada	27	BosStent
Consoli et al.	2024	Retrospective	Canada	12	BosStent
Other stents (exclusively)				Total Patients: 64, Studies: 3	
Koovor et al. [77]	2018	Retrospective	USA	16	Acculink stent (Abbot)
Gorijan [78]	2023	Retrospective	USA	36	LifeStar stent (Bard PV)
Yan [79]	2019	Retrospective	China	12	Sinus-SuperFlex-635 stent (Optimed)
**Multiple stents were used in one study without specifying the numbers for each**				**Total Patients: 619, Studies: 11**	
Liu et al. [80]	2019	Retrospective	China	88	Precise (~80%), Xpert, Wallstent, Solitaire, Protégé
Raper et al. [81]	2018	Retrospective	USA	50	Precise, Protégé Everflex, Zilver biliary
Kumpe et al. [82]	2012	Retrospective	USA	18	Precise, Acculink, or Zilver
El Mekabaty et al. [83]	2021	Retrospective	USA	104	Precise stent (5–10 mm × 40 mm) or Acculink
Song [84]	2025	Retrospective	China	104	Precise or Wallstent
Raygor [85]	2025	Retrospective	USA	96	Zilver or Precise
Levitt [86]	2017	Retrospective	USA	9	Zilver, Wallstent, or Protégé
Teleb et al. [87]	2015	Prospective	USA	18	Wallstents (most used), Express, Smart, Xpert stents
Aguilar-Pérez M et al. [88]	2017	Retrospective	Germany	51	Wallstent, Protégé, Solitaire, balloon-expandable stents
Xu [89]	2024	Retrospective	China	64	Wallstent or Protégé
Smith et al. [90]	2017	Retrospective	USA	17	Wallstent, Precise or/and Zilver
**Unspecified**				**Total Patients: 127, Studies: 6**	
Silva et al. [91]	2017	Retrospective	Brazil	7	Self-expanding stent
Boddu et al. [92]	2016	Prospective	USA	29	Self-expanding stent (8–10 mm × 20–60 mm)
Bussière M et al. [93]	2010	Retrospective	Canada	13	Balloon-expandable or self-expanding stent
Cheng [94]	2023	Prospective	China	16	Self-expanding stent
Ahmed et al. [95]	2011	Retrospective	Australia	52	Self-expanding Stent
Donnet et al. [96]	2008	Retrospective	France	10	Self-expanding stent
**Not Reported (NR)**				**Total Patients: 435, Studies: 7**	
Patsalides et al. [6]	2019	Prospective	USA	50	NR
Goodwin et al. [97]	2014	Retrospective	USA	15	NR
Higgins et al. [98]	2003	Retrospective	UK	12	NR
Iyer [99]	2024	Retrospective	USA	206	NR
Wang et al. [100]	2022	Prospective	China	101	NR
Tanaka [101]	2025	Retrospective	Australia	18	NR
Abdalkader [102]	2025	Retrospective	USA	33	NR

**Table 2:** Preclinical performance of stents used for venous sinus stenosis stenting. This table lists the stents by name and summarizes available animal study data (often from other vascular or non-vascular applications). N/A: not applicable.

Stent	Animal Studies	Type of Study	Species	No.	Implantation Site	Findings	Ref.
**Precise**	Yes	Published articles	Minipig	14 stents	Carotid and subclavian arteries as a reference to evaluate a novel stent (E-volution).	Pig carotid model: No thrombosis; ~34% area stenosis at 4 weeks (max neointima ~0.5 mm); complete endothelialization by 28 days; performance equivalent to reference carotid stents.	[103]
			Pig	6 stents	Internal mammary vein as a reference to assess the safety of a novel self-expanding braided venous stent (VIVA).	Significant increase in venous diameter following deployment of the Precise stent, indicative of its higher radial force; however, this did not translate into increased vessel injury, thrombosis, or delayed healing compared to the test group.	[39]
		FDA testing	Pig (acute study), Dog (chronic)	23 Precise stents for acute porcine study, 63 stents with similar design (S.M.A.R.T. stents) for chronic canine study	Carotid, subclavian, maxillary, iliac, femoral arteries.	Rated excellent, good, or acceptable for stent performance and mechanical characteristics.	[104]
**Protégé**	Yes	Published articles	N/A	N/A	N/A	Bench study: slightly less flexible, more radial force than Precise stent	(Bench [105])
		FDA testing	Pig, dog	39 stents	Carotid artery (15 stents, canine; 6 stents, pig) iliac artery (15 stents, canine; 3 stents, pig), and subclavian artery (9 stents, pig).	Successful tracking and deployment. Within 6 months, no histology results from stenosis.	[106]
**Zilver Stent**	Yes	Published articles	Pig	72 stents	Each animal received two non-overlapping paclitaxel-coated Zilver stents in the left iliofemoral artery and two in the right iliofemoral artery.	Within 3 months, vessels with both paclitaxel-coated and bare-metal stents showed comparable, complete healing.	[107]
			Pig	16 stents	Zilver PTX stents were implanted in the iliofemoral arteries and were compared with another paclitaxel-eluting stent.	Greater vessel healing and less drug impacts were found in the Zilver PTX group.	[108]
			Pig	40 stents	Each animal underwent balloon inflation in both iliofemoral arteries using a drug-coated balloon on one side and an uncoated balloon on the other. A Zilver PTX drug-eluting stent was deployed across each previously ballooned region (1 stent per vessel, 2 stents per animal).	No significant differences in the safety parameters, including inflammation and endothelial cell loss, were observed when comparing the safety of Zilver PTX drug-eluting stents following drug-coated balloon (DCB) angioplasty and conventional balloon angioplasty (BA).	[109]
			Pig	12 stents	Polymer-free paclitaxel-eluting Zilver PTX stents were tested on a familial hypercholesterolemic swine model of femoral restenosis and compared to a bare-metal (Innova) and a new-generation fluoropolymer-based paclitaxel-eluting stent (Eluvia).	The Zilver PTX stent showed moderate inhibition of neointimal hyperplasia compared to a bare-metal stent, but was significantly outperformed by the fluoropolymer-based Eluvia stent.	[110]
			Pig	6 stents	A piglet small intestinal submucosa (SIS) sandwich Zilver endograft was used for experimental transjugular intrahepatic portosystemic shunt (TIPS) creation.	The piglet SIS sandwich Zilver stent graft was found to offer only limited TIPS patency.	[111]
			Pig	6 stents	A flexible sandwich Zilver stent graft with small intestinal submucosa was used for the creation of an intravascular ultrasound (IVUS)-guided direct intrahepatic portocaval shunt (DIPS).	The DIPS was found to be either severely stenosed or occluded at 4 weeks.	[112]
			Sheep	18 stents (total)	Six externally SIS-covered endografts, 6 internally SIS-covered endografts, and 6 bare stents were used. The stents were placed in the balloon-injured external iliac arteries via the carotid approach.	Bare-metal and externally SIS-covered Zilver stents showed excellent biocompatibility, with 100% patency, minimal neointimal hyperplasia, and complete endothelialization by 3 months. In contrast, internally SIS-covered stents performed poorly.	[113]
			Sheep	12 stents	Six bare Zilver nitinol stents, six Zilver stents covered with SIS, and six Palmaz stents covered with polytetrafluoroethylene were implanted in the balloon-injured femoral arteries of nine female sheep.	All of the bare stents and SIS-covered endografts remained patent throughout the follow-up period, but both developed progressive eccentric intimal hyperplasia, which was more pronounced in SIS-covered endografts at 6 months.	[114]
		FDA testing	Domestic and miniature pigs	413 stents	Not reported.	The results demonstrate no safety issues, complete vessel healing, no negative sequelae, and no regional or systemic effects associated with Zilver PTX stents.	[115]
**Wallstent**	Yes	Published articles	Sheep	24 stents	Stents were placed on the iliac veins bilaterally for each animal.	The Wallstent was used as a reference in the control group and demonstrated lower maximal radial resistive force compared to the novel stent, complete endothelialization, no thrombosis, and stenosis rates of ~ 32% at 90 days.	[116]
			Sheep	8 stents	One stent was implanted in the iliac artery of each animal.	The Wallstent was used as a reference and demonstrated the lowest neointimal area and minimal inflammation compared to covered stent grafts, no thrombosis, and the highest endothelialization, with mature, spindle-shaped cells.	[117]
			Sheep	14 stents	Both a Wallstent and a nitinol stent were implanted on opposite sides of iliac arteries.	The Wallstent exhibited significantly less neointimal thickness than the nitinol stent at both 1 and 4.5 months.	[118]
			Pig	3 stents	One stent was implanted in the iliac artery of each animal.	Wallstent implantation led to flattened compliance, diameter pulsatility, and relative pulsatility within the stent, along with increased stiffness, elastic modulus, and wall stress.	[119]
			Pig	13 stents	A total of 50% iliac artery stenoses was created using resorbable ligatures in 13 pigs. After 30 days, PTA was performed bilaterally, with an additional stenting procedure conducted on one side.	Wallstent implantation after angioplasty led to significantly increased platelet and neutrophil deposition compared to angioplasty alone, indicating a notable acute thrombogenic response.	[120]
			Pig	4 stents	Wallstents were implanted into common iliac arteries as a reference for novel drug-eluting stents.	Wallstents exhibited the largest luminal diameter and area and low neointimal thickness, with mild vascular injury and minimal inflammation.	[121]
			Miniature pig, dog	5 stents in total (3 in dogs and 2 in pigs)	Each animal received one Wallstent in one iliac artery.	All Wallstent-treated arteries remained patent at follow-up (45 days in dogs, 32 days in pigs).	[122]
			Pig	5 stents	Each animal received one Wallstent on a common carotid artery and one Precise stent on the contralateral side.	Wallstent implantation led to significant luminal narrowing for all segments (proximal, mid, distal) and was associated with elevated duplex ultrasound velocities, indicating mechanical stiffening and a compliance mismatch.	[123]
			Pig	6 stents	A Wallstent was placed in a novel porcine model of abdominal aortic aneurysms, created using glutaraldehyde-tanned bovine jugular vein interposition grafts.	The Wallstent produced a favorable hemodynamic environment, reducing the pulsatility and size, with partial thrombosis of the sac and high endothelialization rates.	[124]
			Dog	6 stents	Each animal received one silicone-covered stent (Permalume; Boston Scientific Vascular), in the right iliac artery and one Wallstent (Boston Scientific Vascular) of the same diameter and length in the left iliac artery.	Angiography demonstrated no stenoses or occlusions. Histopathology showed no tissue atrophy, necrosis, gross infection, or inflammation. Bare-metal Wallstents demonstrated a smooth glistening neointima that had formed uniformly throughout the lumen.	[125]
			Dog	13 stents	In 7 dogs, Wallstents and Palmaz stents were implanted in contralateral vessels. Six dogs received both iliac and femoral stents. One dog received only iliac stents.	All stented arteries remained patent, with modest neointimal growth and luminal narrowing, comparable to Palmaz stents. Histology showed moderate medial atrophy and some wall trauma from wire ends.	[114]
			Dog	5 stents	Various self-expanding stents, including Wallstents, were endovascularly placed in common carotid arteries.	The SMART stent showed the greatest expansibility. The thickness of the neointimal coverage was more prominent with the SMART stent than the Wallstent.	[126]
			Dog	1 stent	One stainless-steel Wallstent was coated with autologous vein grafts and placed in the carotid–jugular AVF model.	Deployment was technically challenging due to the rigid, sharp steel filaments, which penetrated the vein graft.	[127]
			Dog	8 stents	Stents were placed into both common carotid arteries. In each CCA, two stents were deployed; a stent of the appropriate diameter was implanted distally and, an oversized stent, proximally.	Easy Wallstents, whether normally sized or oversized by 30–40%, preserved 94–96% of the luminal diameter after 4 months, with no significant difference in neointimal hyperplasia compared to appropriately sized stents.	[128]
			Dog	6 stents	A model of end-to-end anastomosis between iliac arteries and polytetrafluoroethylene grafts was developed; a Wallstent was placed across the anastomosis and the opposite limb served as a control.	Wallstents reduced hyperplasia at the anastomotic site, but caused a significant increase in intimal thickening at the proximal stent edge. All stented grafts remained patent.	[129]
			Rabbits	5 stents	Stents were placed in the infrarenal aorta of 13 New Zealand white rabbits.	The Wallstent significantly reduced local arterial wall compliance and distensibility.	[130]
		FDA testing	N/A	N/A	N/A	N/A	
**Acculink**	Yes	Published articles	Sheep	4 stents	Eleven stents of three types were placed in ovine carotid arteries. Precise stents were deployed three times, while Acculink and X-Act stents were placed four times each.	Stent design can affect Doppler readings. The X-Act stent may cause the Doppler to overestimate the blood velocity, leading to potential misinterpretation of restenosis, while the Precise and Acculink stents did not encounter this problem.	[131]
		FDA testing	Pig	90 stents	Stents were implanted in the carotid arteries of a non-atherosclerotic swine model.	Implanted stents did not elicit acute or chronic thrombosis, adverse inflammatory reactions, or excessive neointimal proliferation.	[132]
**Xact/Xpert**	Yes	Published articles	N/A	N/A	N/A	N/A	
		FDA testing	Pig	44 stents	Carotid and iliac arteries.	Good outcomes in terms of safety and performance.	[133]
**LifeStent/LifeStar**	Yes	Published articles				Pig SFA: LifeStent showed slightly higher neointima vs. the competitor (9–12% vs. 5–8% area, ns) in the 30 d study. Swine iliac arteries patent at 6 months; neointima ~0.3 mm. Animal models of high vs. low radial force suggest high COF stents (like LifeStent) might induce more IH if oversized.	
		FDA testing	Pig	14 pigs (the number of stents not specified)	The stents were implanted in various peripheral vasculatures, including femoral and iliac arteries.	The vessel patency was high and the wall response to the stent was minimal. The results support the safety and expected performance of the stent.	[134]
**Casper/Roadsaver**	Yes	Published articles	N/A	N/A	N/A	N/A	
		FDA testing	Pig	48 stents	Chronic studies: Stents were placed in various locations of the porcine carotid and subclavian arteries.	The results of the chronic animal study demonstrated that the stent and delivery system performed as expected and demonstrated the safety of the device for use in humans.	[135]
			Dog	10 stents	Acute animal study: Stents were implanted in one canine animal in various target vessels and locations.	The results of the acute animal study demonstrated that all of the devices performed successfully.	[135]
**Enterprise/Solitaire AB**	Yes	Published articles	Pig	6 stents	Each animal received 1 Enterprise and 1 Pipeline embolization device contralaterally, across the carotid bifurcation (PED).	The results disclosed no difference in thrombogenicity between the two devices, with a thromboembolic event rate of 33.3% for both.	[136]
			Pig	18 stents	In a swine model of acute ischemic stroke, the Solitaire AB/FR stent retriever was compared to a new stent retriever (the Aperio) targeting the subclavian and carotid arteries.	Both devices achieved 100% recanalization. No device-related complications were reported. The results indicated no significant difference between Aperio and Solitaire in terms of performance.	[137]
			Pig	4 stents	6 PED Flex devices, 6 PED Shield devices, and 4 Solitaire AB devices were implanted in the carotid arteries of 4 pigs (2 stents per vessel).	The neointimal area and ratio were significantly lower in the Solitaire group compared to the PED Flex and PED Shield devices. Solitaire recorded no thrombosis events during the early single antiplatelet therapy phase, whereas the PED Flex group experienced high early thrombosis rates.	[138]
		FDA testing	N/A	N/A	N/A	N/A	
**River Stent**	No						
**Viva stent**	Yes	Published article	Pig	9 stents	Implantation in the mammal vein; comparison to 6 Precise stents.	No thrombus formation was observed. CT venography confirmed vessel patency, absence of stent migration, and complete structural integrity. Histopathology revealed a mild, expected foreign body reaction at 30 days that had resolved by 180 days, and an increased luminal diameter and reduced wall thickness at 180 days.	[39]
**BoStent**	No						

**Table 3:** Proposed solutions for preclinical testing of venous sinus stenting devices.

Purpose/Tier	Species and Target Site	Access/Condition	What to Test	Key Endpoints	Key Ref.
**Deployment and healing in a dural sinus (foundational)**	Sheep—SSS	Well-described venography; chronic in sinus implantation feasible despite smaller diameters.	Stent deliverability, apposition, endothelialization.	Patency, neointima, inflammation grading, endothelial coverage at 3–6 months.	[26,27]
**Access feasibility and intracranial sizing**	Pig—SSS	Percutaneous SSS catheterization demonstrated.	Sizing rules, OCT/IVUS guidance.	Intraluminal imaging quality; safe navigation.	[29,31]
**Create venous hypertension (disease construct)**	Baboon or Pig—dural outflow	Stepwise partial occlusion proximal to petrosquamous sinus.	Reproducible gradient and ICP elevation without infarction.	Sustained ICP/venous pressure rise; neuro intact survival.	[22]
**Disease + treatment (efficacy model)**	Baboon or Pig—chronic TS stenosis→stenting	Gradual TS occlusion to induce venous gradient, then stent application.	Hemodynamics and ICP response to stent.	Trans-stenotic gradient; ICP correction; venography.	[22,158]
**Peripheral vein first-stage design screening**	Sheep IVC/iliac; pig jugular/iliac; rabbit paired iliac; pig internal mammary vein	Long-term survival; sizes in regard to human transverse sinus; paired control possible.	Design variables (length, ring spacing, metal-to-vein ratio), oversizing tolerance.	Patency, neointima vs. design; migration risk with oversizing.	[34,38,40,44]
**Small animal mechanistic screening (rapid)**	Rat IIH (obese ± androgen) ± reversible venous stenosis	Telemetric ICP where feasible; transient sinus occluder to simulate “pre-stent” environment.	ICP/CSF dynamic changes.	ICP drop; improved ICP pulsatility; CP/optic nerve markers.	[8,9,14]

## Data Availability

No new data were created or analyzed in this study.

## Abbreviations

The following abbreviations are used in this manuscript:

ALS	Amyotrophic lateral sclerosis
AQP1	Aquaporin-1
BCI	Brain–computer interface
CFR	Code of Federal Regulations
COF	Chronic outward force
CSF	Cerebral spinal fluid
CT	Computed tomography
CVST	Cerebral venous sinus thrombosis
FDA	U.S. Food and Drug Administration
GLP	Good Laboratory Practice
ICP	Intracranial pressure
IIH	Idiopathic intracranial hypertension
IVC	Inferior vena cava
NKCC1	Na^+^-K^+^-2Cl^−^ cotransporter
OCT	Optical coherence tomography
PTX	Paclitaxel
RF	Radiofrequency
SFA	Superficial femoral artery
SSS	Superior sagittal sinus
TIPS	Transjugular intrahepatic portosystemic shunt
VRD	Vascular reconstruction device
VSS	Venous sinus stenting
